# Who will drop out of voluntary social health insurance? Evidence from the New Cooperative Medical Scheme in China

**DOI:** 10.1093/heapol/czab017

**Published:** 2021-05-08

**Authors:** Mingming Xu, Wei Yang

**Affiliations:** Department of Economics and Management, Karlsruhe Institute of Technology, Karlsruhe, Kronenstraße 34, 76133 Karlsruhe, Germany; Department of Global Health and Social Medicine, Faculty of Social Science and Public Policy, King’s College London, 3.09 Bush House NE, Strand, London WC2R 2LS, UK

**Keywords:** Public voluntary health insurance, drop out, the New Cooperative Medical Scheme, China

## Abstract

Although public voluntary health insurance (VHI) has been adopted in many low- and middle-income countries to improve access to care for the population, a common issue with VHI is its high dropout rate. Using the New Cooperative Medical Scheme (NCMS)—a public VHI in China—as a case study, this article employs a fixed-effects negative binomial regression model combining the difference-in-difference-in-differences approach and multivariate distance matching to examine the factors associated with dropping out and the impact of dropout on outpatient care utilization among middle-aged and older people in rural China. Drawing data from the China Health and Retirement Longitudinal Study, our results showed that healthy people, vulnerable people and people who use less healthcare tended to drop out. Dropout had a significant negative impact on outpatient care utilization, especially for those with worse health statuses and those living in poorer provinces. We also found that the impact of dropout on outpatient utilization was more pronounced at secondary and tertiary hospitals than at primary care clinics. We urge policymakers to rethink the design of the NCMS by waiving premiums for the most vulnerable people.

**KEY MESSAGES**

- Over 7% of enrollees in New Cooperative Medical Scheme in China chose to drop out.
- The healthy and the vulnerable tended to drop out.
- Drop out had a significant negative impact on outpatient care utilization, especially for those with worse health status and living in poorer provinces.
- The impact of dropout on outpatient utilization is more pronounced at secondary and tertiary hospitals than at primary care clinics.

## Introduction

Social health insurance (SHI) is one of the commonly used mechanisms for raising revenue and pooling funds to finance health care. In many high-income countries, working-age adults are obliged to be enrolled in an SHI. SHI will usually cover a significant proportion of their medical bills when they seek care. In many low- and middle-income countries (LMICs), with a large number of people in the informal economy, the enforcement of a compulsory SHI can be difficult and costly. Some countries have made SHI voluntary to encourage enrolment so that enrolled households that would otherwise incur significant health costs will have some degree of financial protection when seeking care.

A common issue with voluntary health insurance (VHI) is adverse selection, where insurance draws only high-risk individuals who self-select into the scheme due to potentially high use of healthcare. This limits the scope for risk pooling, undermines financial sustainability, and leads to growing premiums and imbalance in health risks in the VHI scheme (World Health Organization, 2013). Studies also show that the VHI may exclude a large proportion of the population who are likely to be poor and vulnerable. People may choose not to be enrolled because of the high insurance premiums. This, in turn, will harm the goal of SHI to improve equal access to care by redistributing health care resources (World Health Organization, 2005).

This study focuses on a public VHI adopted by the Chinese government. In 2003, the New Cooperative Medical Scheme (NCMS) was established to cover the rural population following the failure of its old version (State Council China, 2002). The former version of the NCMS was launched in rural areas in the late 1950s and covered the rural population in the mid-1970s. Based on the people’s commune system—the largest collective unit in the 1960s—the scheme was offered to the participants free of charge, and the participants did not need to make individual contributions to the scheme. However, this scheme was dissolved in the late 1980s together with the people’s commune system. As a consequence, the rural population in China was not covered by any SHI or health financing programmes for nearly two decades (Hillier and Shen, 1996; Soares, 2009; Wagstaff *et al.*, 2009b; Blumenthal and Hsiao, 2015). The NCMS, according to the insurance policy document published in 2003, was defined as a mechanism through which the state should provide equitable access to healthcare and financial protection against catastrophic illness for all rural populations, and it should be offered to all rural populations irrespective of individual characteristics such as gender, job status, education and level of wealth (State Council China, 2003). It is a voluntary scheme financed by participants’ contributions and heavy government subsidies (approximately 80%) (Blumenthal and Hsiao, 2015; Hipgrave and Mu, 2015; Meng *et al.*, 2015; Yu, 2015). Within less than 5 years of its inception, the NCMS reached >90% of the rural population. This initial success of the broad population coverage should be credited to local governments’ promotional efforts since the central government has set NCMS enrolment as an important administrative assessment indicator and the low premiums and high subsidies (Lei and Lin, 2009; Hao *et al.*, 2010; Babiarz *et al.*, 2012). In 2019, individuals were required to pay a premium of RMB250, and the government subsidized a flat rate of RMB520 (Chinese National Healthcare Security Administration, 2019). Over the past decade, the scheme has expanded its benefits package and increased its reimbursement rates for services, but enrolment in the scheme remains voluntary for the population.

A number of studies have examined factors influencing people’s decision to enroll in a public VHI. These studies identified individual or household factors such as age, household income and education level as influencing factors that drive enrolment (Jowett, 2003; Nguyen and Knowles, 2010; Khalid and Serieux, 2018; Oraro *et al.*, 2018). Most of these studies have taken analytical approaches that reflect the established institutional and socioeconomic conditions in their specific study contexts—Africa or Southeast Asia—which may not be applicable to China. A small number of studies have explored the voluntary nature of SHI in China, but none of these studies examined the factors influencing people’s decision to drop out, and it is not clear how dropping out affects the use of health care resources (Wang *et al.*, 2006; Yang and Wu, 2015). Moreover, there remains a dearth of literature on how the voluntary nature of SHI affects the utilization of health care among rural older people who are likely to have vulnerable demographics or have a lower socioeconomic status than younger people.

Based on the discussion above, this study seeks to examine the characteristics of dropout in this VHI programme and the effects of dropout on outpatient utilization using the NCMS from China as an example. Drawing data from the China Health and Retirement Longitudinal Study (CHARLS) 2013 and 2015, this study uses a logistic regression to explore the characteristics of older people who drop out and a fixed-effects negative binomial regression with the difference-in-difference-in-differences (DDD) approach (Wooldridge, 2013; Imbens and Wooldridge, 2007) and multivariate distance matching (MDM) to examine the impacts of dropout on outpatient care utilization. By analysing the issue of dropping out of VHI in China, this study aims to gain a thorough understanding of the retainment of VHI membership and the associated effects on healthcare utilization. The study results have significant implications for policymakers in LMICs where similar schemes have been or will be implemented.

## Public voluntary health insurance and dropout

Dropping out seems to be inevitable for public VHI due to the issue of adverse selection (Akerlof, 1970; World Health Organization, 2013). For most public VHIs, a flat rate premium, instead of a risk-based premium, is usually charged to ensure fairness in health financing and practical implementation. Since a flat rate premium does not always reflect the risk profile of young and healthy individuals, these individuals may choose not to participate or drop out of insurance (Rothschild and Stiglitz, 1976). Empirically, studies have found consistent evidence with this theoretical inference and concluded that dropout is not uncommon for VHI (Mladovsky, 2014; Atinga *et al.*, 2015; Boateng *et al.*, 2017; Herberholz and Fakihammed, 2017; Iqbal *et al.*, 2017). The majority of these studies were conducted in LMICs and in the setting of community-based health insurance, which is often voluntary and requires people to pay flat rate premiums (Donfouet and Mahieu, 2012; World Health Organization, n.d.). A study conducted in India shows that up to 80% of the initial participants in a VHI programme did not maintain their membership in the following year, and only those who had benefited from the scheme tended to renew the membership (Panda *et al.*, 2016). Studies also show that people with worse health status and high health risks were less likely to drop out in Eastern Sudan (Herberholz and Fakihammed, 2017). This leads to the issue of adverse selection that insurance may have a large proportion of participants who have high health risks and are potentially more likely to use healthcare services. When a significant proportion of participants belong to the high-risk category, insurance funds may have to either raise premiums or reduce benefits, and this may again lead those who perceive their risks to be low in relation to the premium/benefits ratio to drop out (Mossialos and Thomson, 2004). Some researchers have indicated that one of the greatest challenges for a VHI programme is the high dropout rate, and others have raised concerns about the sustainability of the financing of a VHI programme (Dong *et al.*, 2009; Mebratie *et al.*, 2015).

In addition to adverse selection of VHI, the literature so far has demonstrated that the dropout or enrolment of a VHI programme may also be associated with other socioeconomic factors. For instance, Khalid and Serieux (2018) found that wealthier and more educated individuals are more likely to buy VHI in Ghana. Similar results were demonstrated by a study conducted in Malawi (Abiiro *et al.*, 2016). Another study examining insurance updates in northwestern Cameroon found that households with no income or poorly educated household heads had a poor understanding of health risks and low enrolment in VHI (Oraro *et al.*, 2018).

A number of studies have investigated the impacts of VHI enrolment on healthcare utilization. Supakankunti (2002) found that health service utilization was significantly increased after the VHI enrolment in Thailand. Similar findings were demonstrated in Vietnam and Ghana, where enrolment in a VHI was strongly associated with higher outpatient and inpatient visits (Nguyen, 2012; Khalid and Serieux, 2018). Studies on the NCMS in China have so far demonstrated mixed results. Yu *et al.* (2010) found no significant association between enrolment and outpatient care utilization, Li and Zhang (2013) found limited positive impacts on healthcare utilization, and Zhang *et al.* (2016) found that there were more doctor visits among the insured than the uninsured. However, after combining DID and matching method, Wagstaff *et al.* (2009a,b) found that both outpatient and inpatient utilization significantly increased after enrolment.

Although existing studies have provided some evidence regarding VHI in LMICs, empirical studies on VHI in China are rather scarce. Most studies conducted in the context of China focus on VHI uptake rather than dropping out. It is not clear whether participants will continue to participate in VHI, what leads them to drop out, and how dropping out affects the use of outpatient services—the most frequently used care option for rural residents. Furthermore, limited research has investigated these issues among middle-aged and older people who constitute a significant proportion of the rural population and often tend to have higher health needs than the younger population. Findings on this segment of the population are likely to inform the design and implementation process of the public VHI programme in rural China.

## Methods

### Data source and sample selection

This study uses data from CHARLS 2013 and 2015 (CHARLS, n.d.). CHARLS collects a nationally representative sample of middle- and old-aged residents in China and provides individual-level panel data on health, socio-economic status and social and family networks every 2 years (Zhao et al., 2013). Data from the most recent waves (2013-Wave 2 and 2015-Wave 3) were used in the study. Wave 1 (2011) was excluded since its inclusion would reduce the number of eligible individuals due to attrition and new enrolment. Since the analysis is based on two panels, this study adopted balanced data to detect the variation within groups. We also merged data from CHARLS with economic data (provincial GDP per capita) from the National Bureau of Statistics of China (2019).

Chinese health insurance schemes mainly consisted of Urban Employee Basic Medical Insurance (UEBMI), Urban Resident Basic Medical Insurance (URBMI), the NCMS and Government Medical Insurance (GMI) before 2016, among which URBMI and NCMS are VHI (Meng *et al.*, 2015). The State Council officially released a nationwide regulation on integrating the NCMS and URBMI into Urban and Rural Resident Medical Insurance (URRMI) in 2016, but the progress has been uneven, and some individuals in our sample were enrolled in URRMI even before 2016. Table 1 shows the insurance type of individuals enrolled in both years after excluding 285 participants (1.5%) in 2013 and 845 (4.0%) in 2015 due to missing data on insurance type. Among the 15 669 individuals, the majority were enrolled in the NCMS (over 70%). The sample sizes for the URBMI and URRMI participants were relatively small (both <5%). Therefore, considering the heterogeneity of participants in the NCMS, URBMI and URRMI, we selected the NCMS as an example of VHI in our study. As shown in the 3rd row of Table 1, the majority of the participants in the NCMS in 2013 did not change their insurance type in 2015 while some dropped out (with no insurance). In order to explore the impact of dropout, our study sample only included those who were enrolled in the NCMS in 2013 and dropped out in 2015 (treatment group) and those who were enrolled in the NCMS in both years (control group). We finally selected 22 982 observations across two waves in the study by further excluding people aged below 45, among which 1710 were in the treatment group. Since participation in the NCMS is household-based, the participants may not necessarily be rural residences (approximately 95.7% are rural). Table 2 presents the summary statistics of the sample. The distribution of the number of doctor visits for our sample is shown in Supplementary Appendix S1. Overall, 79.1% of observations had zero doctor visits.

**Table 1:** The number of individuals with each insurance type across two waves for 15 669 individuals enrolled in both CHARLS 2013 and 2015

2015 2013	UEBMI	URBMI	NCMS	URRMI	GMI	None	Total (%)
UEBMI	1181	89	43	9	208	137	1667 (10.6)
URBMI	105	419	87	29	15	74	729 (4.7)
**NCMS**	**82**	**107**	**10 718**	**267**	**52**	**879**	**12 105 (77.3)**
URRMI	9	45	214	22	2	24	316 (2.0)
GMI	111	8	11	2	110	17	259 (1.7)
None	28	46	283	10	15	211	593 (3.8)
Total (%)	1516 (9.7)	714 (4.6)	11 356 (72.5)	339 (2.2)	402 (2.6)	1342 (8.6)	15 669

**Table 2:** Descriptive statistics of 22 982 observations in 2013 and 2015

	2013, mean (SD)		2015, mean (SD)	
	Drop-out group	Control group	Drop-out group	Control group
Doctor visits				
All health facilities	0.42 (1.07)	0.52 (1.54)	0.38 (1.30)	0.47 (1.52)
Secondary and tertiary hospitals	0.14 (0.61)	0.13 (0.67)	0.14 (0.78)	0.13 (0.67)
Primary care clinics	0.24 (0.82)	0.34 (1.32)	0.22 (0.96)	0.31 (1.28)
Equivalent income, RMB				
Quartile (25%)	587.9	750.6	0	115.5
Quartile (50%)	2840.6	4780	1000	1500
Quartile (75%)	12 990.4	14 433.8	9263.1	11 547.0
Marital status, %				
The married	77.2	88.4	75.4	86.6
The single	22.8	11.6	24.6	13.4
No. of chronic diseases	1.22 (1.29)	1.28 (1.33)	1.27 (1.31)	1.41 (1.41)
Self-perceived health status, %				
Excellent	4.45	4.69	5.04	5.96
Very good	9.26	10.9	11.1	9.97
Good	31.9	31.1	26.7	30.3
Fair	34.9	36.8	37.2	36.2
Poor	19.5	16.6	20.0	17.6
Age	62.2 (10.6)	59.5 (9.39)	64.2 (10.7)	61.4 (9.39)
Gender, %				
Male	40.6	46.9	40.6	46.9
Female	59.4	53.1	59.4	53.1
Education attainment, %				
No education	43.6	29.9	43.2	30.0
Elementary, middle school	52.5	63.4	46.7	54.6
High school and above	3.86	6.67	10.2	15.4
Occupation				
Agricultural work	48.4	53.6	44.8	47.7
Employed	11.5	15.0	9.41	17.2
Self-employed	7.45	9.39	8.52	8.38
Retired/receded	2.02	1.45	2.04	1.34
Unemployed	30.7	20.6	35.2	25.3
Prop. of drop-out, %	7.44			
*n*	855	10 636	855	10 636

SD, standard deviation (in parentheses).

### Variable specifications

We include the following dependent variables in the study. First, whether a person has dropped out from the NCMS is used to explore the characteristics of people who drop out. This is a binary variable with 1 indicating the respondent participated in the NCMS in 2013 but not 2015 and 0 indicating the respondent participated in both years. Second, the number of outpatient doctor visits during the last month is included. In order to explore the impact of dropout on visits at different levels of health facilities, we conducted analyses using primary care clinics, secondary and tertiary hospitals and all health facilities.

We include a number of health needs and non-needs variables in the analyses. Health needs variables include the number of chronic diseases and self-perceived health status. These variables are likely to impact people’s decision to participate in insurance and healthcare utilization (Andersen and Newman, 1973; de Boer *et al.*, 1997). We further controlled for age, gender, marital status, region and three socioeconomic status indicators: equivalent income, education and occupation. Equivalent income is equal to household income divided by the square root of the household size (OECD, 2011). Self-perceived health status (1. Excellent, 2. Very good, 3. Good, 4. Fair and 5. Poor), education attainment (1. No education, 2. Elementary or middle school and 3. High school and above) and occupation (1. Agricultural work, 2. Employed, 3. Self-employed, 4. Retired/receded and 5. Unemployed) are categorical variables. Marital status comprises a binary variable—married/cohabiting as 0 and single as 1. We assign 28 provinces, where the samples come from, into three regions according to the order of provincial GDP per capita in 2015—Region 1 indicates provinces with the highest GDP per capita, Region 2 is the provinces with the middle GDP per capita and Region 3 is the provinces with the lowest GDP per capita (Zhu and Österle, 2017; Yang and Tan, 2019).

### Empirical strategies

The empirical strategies used in the article involve two parts. We first use a logistic regression model to examine the characteristics of those who are likely to drop out from the NCMS. We then use a fixed-effects negative binomial regression model with the DDD approach and MDM method to understand the impacts of dropping out on outpatient visits. All analyses were conducted using Stata V.13.1 (StataCorp, College Station, TX, USA). The MDM was performed using the Stata module KMATCH (Jann, 2017a). We include a detailed discussion on how these strategies are performed.

#### Analysing the characteristics of those who are likely to drop out from the NCMS

A logistic regression is used to understand the characteristics of those who are likely to drop out based on the 2015 data when the dropout occurred. In order to explore whether healthcare use in previous years would influence people’s decision to drop out, we include the number of doctor visits in 2013 in the model. In order to consider the variance across villages, we adjust the standard errors for the 403 village clusters. We estimate the following regression:

$$\left\{ {\begin{array}{*{20}{c}}
{ln\frac{{{p_i}}}{{1 - {p_i}}} = \alpha + \beta {X_i},0 \lt {p_i} \lt 1} \\
{\Pr \left( {Trea{t_i} = 1} \right) = {p_i}}
\end{array}} \right.$$

where *Treat* indicates whether individual *i* drops out in 2015 (dropout when *Treat* = 1), *p* is the probability of dropping out and $\frac{p}{{1 - p}}$ is the odds of dropping out. *X* denotes the covariates, including the number of doctor visits in 2013, the number of chronic diseases, self-perceived health status, age, gender, marital status, region and the three socioeconomic status indicators: equivalent income, education and occupation. *i* indicates an individual.

#### Analysing the impacts of dropout on outpatient care utilization

We use the DDD with MDM to analyse the impacts of dropping out on outpatient care utilization. We test the pre-treatment parallel trend assumption for the DDD approach between the treatment and control groups with data from 2011 and 2013. The results show no significant difference in doctor visits between the two groups, illustrating that a parallel trend is satisfied (Supplementary Appendix S2). A negative binomial regression model is adopted since the dependent variable is a count variable and there is an overdispersion issue (Gardner *et al.*, 1995). Our model is shown as follow:

\begin{equation*} {y_{it}} = \exp \left( {{b_0} + {b_1}Trea{t_i} + {b_2}Pos{t_t} + {b_3}{Z_{it}} + {b_4}\left( {Trea{t_i}{\text{*}}Pos{t_t}} \right) + {b_5}\left( {Trea{t_i}{\text{*}}{Z_{it}}} \right) + {b_6}\left( {Pos{t_t}{\text{*}}{Z_{it}}} \right) + {b_7}\left( {Trea{t_i}{\text{*}}Pos{t_t}{\text{*}}{Z_{it}}} \right) + {X_{it}} + {\nu _i} + {\varepsilon _{it}}} \right) \end{equation*}

where *y* denotes the number of doctor visits. *Treat* denotes the treatment group when *Treat *=* *1 and the control group when *Treat *=* *0. *Post* controls for time fixed effects, which denotes the year after treatment when *t *=* *1 and the year before when *t *=* *0. *Z* denotes either the number of chronic diseases or regions; and *X* denotes the following time-varying covariates, including marital status, the number of chronic diseases, self-perceived health status, provincial GDP per capita, equivalent income, education and occupation. *t* is the year indicator. *V* controls for the individual fixed effects, which includes both observable (such as gender and birth of year) and unobservable fixed effects. *ε* is the idiosyncratic error term. The coefficient of the three-way interaction term $Trea{t_i}{\text{*}}Pos{t_t}{\text{*}}{Z_{it}},\;\;{b_7}$ shows the DDD estimates.

We run a series of sub-group analyses to understand the heterogeneous impacts. Specifically, we ran regressions to explore the heterogeneity among the following sub-groups: (1) People with different numbers of chronic diseases (by adding the interaction term among Treat, Post and chronic diseases). The literature has revealed that without health insurance, people with chronic diseases tend to seek less care, encounter catastrophic health expenditures and have higher mortality (Rice *et al.*, 2005; Sun *et al.*, 2009; Bittoni *et al.*, 2015). (2) People living in different regions (by adding the interaction term among *Treat*, *Post* and region). (3) People utilizing outpatient care at different levels of health facilities: primary care clinics (i.e. township hospitals and village clinics), secondary and tertiary hospitals and all health facilities.

Since dropout can be an endogenous choice made by individuals, the possibilities of outpatient care utilization and other characteristics may not be balanced between the treatment and control groups, thus introducing selection bias. Following the approach by Wagstaff *et al.* (2009a) who adopted DID with a matching method to explore the association between healthcare utilization and self-selected enrolment in the NCMS, we estimate the impacts of dropout by combining the DDD approach with the MDM. By doing so, we could eliminate all the differences in the observables between the two groups. Our estimates would be unbiased if we assume there are no time-varying unobservables (Wagstaff *et al.*, 2009a). The idea of the MDM is to match observations with the ‘close’ distance metric of all covariates by measuring the Mahalanobis distance between two observations in the multivariate space (Rubin, 1979). Thus, MDM can detect all differences in multi-dimensional covariates and help reduce the imbalance until all information in the covariates has been exhausted. The matching algorithm we used is kernel matching, which allows control variables with smaller distance metrics to be given larger weights (Jann, 2017b).

In order to avoid selection bias and balance outpatient care utilization and other characteristics between the treatment and control groups, we matched the two groups on the following variables in the pre-treatment period (2013): doctor visits, marital status, equivalent income, number of chronic diseases, self-perceived health status, gender, age, provincial GDP per capita, education and occupation. The match rates for both groups are approximately 98% (Supplementary Appendix S3). We use the standardized difference instead of the *t*-test to perform the balance diagnostics. The standardized difference is not affected by sample size and allows us to compare the relative balance of variables with different units (Austin, 2009). According to the results, the two groups have been well balanced after matching since all the differences are <0.1, within an accepted level (Rosenbaum and Rubin, 1985) (Supplementary Appendix S4).

## Results

### Who tends to drop out?

The descriptive statistics are presented in Table 2. It shows that 7.44% (855/11 491) of the participants aged 45 and above in the NCMS chose to drop out in 2015. It is similar to the dropout rate in 2013 (7.27%) according to the data in 2011 and 2013. The logistic regression results and odds ratios (ORs) of dropping out are shown in Table 3. We first add all the following three variables, number of doctor visits in 2013, number of chronic diseases and self-perceived health status, in the model (Column 1), and then add each of them separately since we presume that the three factors are correlated (Column 2–4). As shown in Column 1, the results suggested that people with fewer chronic diseases (low-risk individuals) tended to drop out. The results showed that having one more chronic disease was significantly associated with an 11.7% [(1 − 0.883) * 100%] decrease in the odds of dropping out, meaning that people with fewer chronic diseases were more likely to drop out. However, the coefficients of self-perceived health status indicate that people with poor health were more likely to drop out compared to people with excellent health, but this is not statistically significant. Therefore, the choice of dropout seems not to rely on individuals’ self-perceived health status, which is a subjective perception, but to rely more on objective diagnosis (number of chronic diseases). In addition, when people had one more doctor visit in 2013, their odds of dropping out in 2015 decreased by 6.7% [(1 − 0.933) * 100%]. Column 2–4 present consistent results.

**Table 3:** Odds ratios of dropping out from the NCMS in 2015

	Odds ratios of drop-out/standard errors			
	(1)	(2)	(3)	(4)
No. of doctor visits in 2013	0.933** (0.0300)	0.923** (0.0308)		
Equivalent income (ref: Quartile 1)				
Quartile 2	0.780** (0.0936)	0.798* (0.0965)	0.766** (0.0928)	0.814* (0.0967)
Quartile 3	0.810* (0.103)	0.825 (0.105)	0.803* (0.101)	0.847 (0.107)
Quartile 4	0.931 (0.117)	0.950 (0.119)	0.909 (0.114)	0.943 (0.118)
No. of chronic diseases	0.883*** (0.0314)			0.889*** (0.0313)
Self-perceived health status (ref: Excellent)				
Very good	1.215 (0.288)		1.175 (0.259)	
Good	1.099 (0.222)		0.979 (0.189)	
Fair	1.312 (0.271)		1.118 (0.224)	
Poor	1.426 (0.319)		1.137 (0.244)	
Education attainment (ref: No education)				
Elementary, middle school	0.732*** (0.0703)	0.722*** (0.0689)	0.727*** (0.0682)	0.721*** (0.0688)
High school and above	0.609*** (0.0922)	0.593*** (0.0887)	0.600*** (0.0906)	0.579*** (0.0875)
Occupation (ref: Agricultural work)				
Employed	0.707** (0.111)	0.682** (0.106)	0.710** (0.111)	0.661*** (0.102)
Self-employed	1.251 (0.180)	1.211 (0.172)	1.217 (0.173)	1.183 (0.167)
Retired/receded	1.829** (0.496)	1.765** (0.477)	1.783** (0.483)	1.747** (0.472)
Unemployed	1.281** (0.126)	1.269** (0.121)	1.210* (0.118)	1.296*** (0.124)
Marital status (ref: The married)				
The single	1.645*** (0.195)	1.662*** (0.196)	1.679*** (0.193)	1.692*** (0.198)
Age	1.016** (0.00643)	1.015** (0.00606)	1.014** (0.00633)	1.015** (0.00608)
Gender (ref: The male)				
The female	1.054 (0.0849)	1.011 (0.0786)	1.025 (0.0803)	1.018 (0.0791)
Region (ref: The middle provinces)				
The richest provinces	1.972*** (0.339)	1.998*** (0.333)	2.020*** (0.341)	1.973*** (0.330)
The poorest provinces	1.506*** (0.210)	1.493*** (0.205)	1.504*** (0.205)	1.467*** (0.198)
Constant	0.0233*** (0.0104)	0.0267*** (0.0112)	0.0261*** (0.0116)	0.0303*** (0.0126)
*n*	11 491	11 491	11 491	11 491

Estimates are derived from logistic regression models. Standard errors are clustered on villages (listed in parentheses). Significance levels: ***P < 0.01; **P < 0.05; *P < 0.1.

We also found that vulnerable people, namely, the poorest people, people with no formal education, retired/receded and unemployed people, single people and old people, were more likely to drop out. The coefficients show that the poorest people were more likely to drop out compared to the other groups, though estimates for some quartiles are not statistically significant based on traditional significance criteria. For people with an above high school education, their odds of dropping out are approximately 0.6 times those of people with no formal education. Compared with people performing agricultural work, employed individuals (OR = 0.7) are less likely to drop out while retired/receded individuals (OR = 1.8) and unemployed individuals (OR = 1.3) are more likely to drop out. The odds of dropping out for single people were approximately 1.6 times that of married people. The probability of dropping out increased significantly with age.

### Impacts of dropout on outpatient care utilization

Table 4 shows the impacts of dropout on outpatient care utilization at all health facilities by presenting the incidence rate ratios (more details are provided in Supplementary Appendix S5, which further includes the estimates of covariates). In Column 1, the DID estimate shows that the number of doctor visits after dropout would be approximately 80% of that before dropout.

**Table 4:** Impacts of dropout on outpatient care utilization at all health facilities

	Incidence rate ratios of doctor visits		
	(1)	(2)	(3)
Treatment * year	0.801* (0.108)	1.192 (0.272)	0.376*** (0.101)
Treatment	0.748 (0.201)	0.410** (0.147)	1.694 (0.948)
Year, 1 (2013, ref)			
Year, 2 (2015)	1.201 (0.158)	1.496* (0.331)	1.348 (0.355)
Treatment * year * No. of chronic diseases		0.735*** (0.0801)	
Treatment * No. of chronic diseases		1.472*** (0.204)	
Year * No. of chronic diseases		0.906 (0.0939)	
Treatment * year * 1.region			2.805** (1.188)
Treatment * year * 2.region (ref)			
Treatment * year * 3.region			2.341*** (0.754)
Treatment * 1.region			0.264 (0.247)
Treatment * 2.region (ref)			
Treatment * 3.region			0.397 (0.264)
Year * 1.region			1.619 (0.655)
Year * 2.region (ref)			
Year * 3.region			0.933 (0.284)
Covariates	Yes	Yes	Yes
*n*	22 982	22 982	22 982

Estimates stem from conditional fixed-effects negative binomial specifications. Coefficients represent incidence rate ratios. Standard errors are in parentheses. Significance levels: ***P < 0.01; **P < 0.05; *P < 0.1.

In Column 2, a three-way interaction term among treatment, year and number of chronic diseases was added to the model in order to explore whether the dropout effect varies among people with different health statuses. The results show that the impact of dropout on outpatient care utilization at all health facilities was significantly related to the number of chronic diseases. Specifically, the number of doctor visits for people with one more chronic disease were 26.5% [(0.735 − 1) * 100%] more negatively impacted by dropout.

We further explored whether the impacts of dropout vary between regions as China has seen uneven economic development among different provinces. In Column 3, Table 4, the estimates of the three-way interaction show that the impacts of dropout on doctor visits significantly depend on the region. In particular, doctor visits in Region 2 were most negatively impacted by dropout [decrease by 62.4%, calculated with (0.376 − 1) * 100%]. Compared with Region 2, the negative impact of dropout on doctor visits in Region 3 with the lowest GDP per capita was lower. When adding the DID estimate and the estimate of the three-way interaction of Region 3, we obtain the dropout effect in Region 3 [(0.376 * 2.341 − 1) * 100% = −12.0%]. This means that people in Region 3 with the lowest GDP per capita would have an 11.9% reduction in doctor visits caused by dropout. Furthermore, people in Region 1 with the highest GDP per capita do not seem to be negatively impacted by dropout. The results show that people in Region 1 would slightly increase their doctor visits due to dropout [5.5%, calculated with (0.376 * 2.805 − 1) * 100%].

As shown in Table 5, we conducted further analyses on outpatient care utilization at primary care clinics and secondary and tertiary hospitals (more details are provided in Supplementary Appendix S6, which further includes the estimates of the covariates). The results suggest that the impacts of dropout occur only at secondary and tertiary hospitals while no significant effects were observed at primary care clinics. After dropping out, people’s doctor visits to secondary and tertiary hospitals significantly decreased by 38.3% [(0.617 − 1) * 100%].

**Table 5:** Impacts of dropout on outpatient care utilization at different health facilities

	Incidence rate ratios of doctor visits	
	Primary care clinics	Secondary and tertiary hospitals
	(1)	(2)
Treatment * year	1.065 (0.189)	0.617** (0.146)
Treatment	0.496** (0.171)	1.595 (0.909)
Year, 1 (2013, ref)		
Year, 2 (2015)	1.096 (0.190)	1.299 (0.297)
Covariates	Yes	Yes
*n*	22 982	22 982

Estimates stem from conditional fixed-effects negative binomial specifications. Coefficients represent incidence rate ratios. Standard errors are in parentheses. Significance levels: **P < 0.05.

## Robustness checks

We conduct the following robustness checks. First, we use equivalent expenditures to replace income as the measure of economic status in the regression model. Expenditures can be a better indicator of economic status for LMICs. We found robust and consistent results for both the factors influencing drop out and the impacts of drop out on doctor visits to all health facilities (Supplementary Appendix S7). Second, we conduct an additional fixed-effects logistic regression by including the uninsured individuals in 2013 in our sample to understand the characteristics of unenrolled individuals. The results are consistent with the main analysis and indicate that people with fewer chronic diseases tend to have no health insurance (Supplementary Appendix S8). Third, we present the results of a logistic regression with adjusted standard error clustering at the household level. We obtained consistent results (Supplementary Appendix S9).

## Discussion

Understanding whether and how VHI addresses gaps in healthcare coverage is important. Using China as a case study, this article offers compelling new findings regarding issues relating to dropping out from a public VHI programme. We first examined adverse selection in the NCMS and found that those with worse health status were more likely to maintain their enrolment status. We then examined whether dropout would cause variations in accessing healthcare and found that the dropout group used less care due to lack of insurance protection whereas the enrolled group tended to use more care.

Specifically, we found that over 7% of the participants in the NCMS chose to drop out. Healthy people, vulnerable people, people who use less care, and people from the richest and poorest regions tended to drop out. According to our findings, we would be reluctant to ascertain the existence of adverse selection though we found healthy people (with less chronic diseases based on objective diagnosis) tended to drop out, because the self-perceived health status (subjective perception) indicates a different direction though insignificant and it is believed that adverse selection should be more associated with subjective perception of illness (Browne and Doerpinghaus, 1993). As for the reason why healthy people tend to drop out, one possible explanation is that the healthy participants may evaluate their health risk as low in relation to the insurance premium and would be reluctant to renew their enrolment (Rothschild and Stiglitz, 1976). Vulnerable people tend to drop out due to unaffordability and a lack of understanding of the NCMS. The value of the premium may be high in relation to their limited disposable income. Therefore, they are also more likely to self-perceive themselves to be low-risk individuals (Brown and Oates, 1987; World Bank, 2000; Stoller and Stoller, 2003). Those with no education and older people are more likely to drop out due to a lack of knowledge of insurance since these people are perceived as slow in obtaining new knowledge (Kadefors and Hanse, 2012; Gorges *et al.*, 2016). The significant regional factor may indicate the variations in the enrolment process in the richest and poorest regions (Barber and Yao, 2010).

We also found that dropout has significant negative effects on overall outpatient utilization, and these findings are in line with existing evidence (Wagstaff *et al.*, 2009a; Li and Zhang, 2013). In addition, our findings showed that people with more chronic diseases were more negatively affected by dropout. One possible explanation is that these people need to visit doctors more frequently, they are more likely to incur greater financial burdens and then they may choose not to seek care if they are not covered by insurance. We also found that dropout only affected outpatient utilization at secondary and tertiary hospitals but not at primary care clinics. This is not surprising as services are often more costly at the secondary and tertiary levels. For those without any insurance coverage, seeking care at secondary and tertiary hospitals may be too expensive to afford.

These considerable regional variations exist in terms of the effect of dropout on outpatient utilization. People from deprived rural villages may encounter greater barriers to access and have fewer financial resources to pay for their treatments. Without the NCMS, these people may be less willing to seek outpatient care, even if they need it. Similar findings have been demonstrated by other studies (Nemet and Bailey, 2000; Arcury *et al.*, 2005). Furthermore, we also found that the people in the richest region would even increase their doctor visits after dropping out, which is perhaps related to the more developed commercial health insurance market in rich provinces (Choi *et al.*, 2018).

This study presents meaningful results in search of a better insurance design to improve access to healthcare for the NCMS participants in China. Several implications can be drawn. First, it is important to establish a more comprehensive mandatory health insurance provision for the rural population that accounts for over 40% of the whole population (National Bureau of Statistics of China, 2019). Voluntary enrolment in the NCMS should only be an interim plan. In order to ensure adequate risk pooling, maintain the sustainability of the NCMS and benefit a large amount of the rural population, an ingenious method of transforming the NCMS into compulsory health insurance should be considered in the long run (Akerlof, 1970; Mossialos and Thomson, 2004; World Health Organization, 2013).

Second, the government should promote the enrolment of vulnerable individuals by increasing the subsidies or waiving insurance premiums. Although the Chinese government has initiated a medical assistance safety net scheme, the scheme only covers a small proportion of the population. Many vulnerable people may still have to pay their medical bills out of pocket. Evidence from other countries has shown that exemptions for people with lower socioeconomic status should be improved; and by improving safety nets for them, basic healthcare access could be guaranteed (Hall *et al.*, 2012; Derbile and Van Der Geest, 2013).

Third, the primary care system should be strengthened, and primary care quality should be improved. Numerous studies have shown that patients often bypass primary health facilities and go to secondary or tertiary hospitals to seek care, even for minor ailments (Li and Xie, 2013; Liu *et al.*, 2018). Although a higher service pricing system at secondary and tertiary hospitals is effective in keeping patients at primary care clinics, it also means that those from socially disadvantaged groups would have fewer choices and may have to use cheap, low-quality services. Therefore, improving primary care quality is vital, especially for vulnerable individuals.

Finally, a fiscal redistribution system should be enhanced to reduce inequities in access to care arising from different regions. In Western and Central China, where the level of economic development remains low, local governments and rural residents have a limited capacity to finance the overall programme adequately. In the more prosperous Eastern and coastal regions, the insurance benefit package is usually much more comprehensive. Using the NCMS as an example, wealthy provinces should be encouraged to improve benefit packages using their own resources; and the central government should ensure a minimum package for the NCMS in all provinces, which should be financed through the fiscal transfer system, that is, to require richer provinces to pay proportionally more (Wagstaff *et al.*, 2009b).

Contemplating the policy implications, we must consider some limitations of this study. First, we have a high enrolment rate (approximately 90%) in the NCMS. We have a relatively smaller sample for the unenrolled group, which can be attributed to various reasons, such as lack of understanding of the scheme and mismanagement of the implementation process. Therefore, based on the current study, the reasons for dropout cannot be totally attributed to better health status. Second, since private pharmacies play an important role as primary caregivers in rural China, there may be a substitution effect for doctor visits when people drop out from the NCMS. However, we cannot detect it based on the available data. Third, we cannot eliminate the bias caused by time-varying unobservables. Suppose an individual has recently visited doctors and is now confident that no more care would be needed in the near future. The unobservable healthcare need has varied with time, and this group of people may select to drop out of the NCMS, but their decreased care use in the near future is not totally due to drop out (Wagstaff *et al.*, 2009a). Last, we are unable to evaluate the impact of drop out on health status since we do not have sufficient data to capture health status changes.

## Supplementary data

Supplementary data are available at *Health Policy and Planning* online.

## Supplementary Material

czab017_Supp
