# Supplementary material for: Who will drop out of voluntary social health insurance? Evidence from the New Cooperative Medical Scheme in China
Source: Health Policy Plan. 2021 May 8;36(7):1013–22. doi: 10.1093/heapol/czab017 (PMC8530158; doi:10.1093/heapol/czab017)
Supplement: czab017_Supp [file czab017_supp.zip › Response letter 190121.docx]

*REVIEW OF RESUBMISSION:* HEAPOL-2020-Sep-0762.R1

**Who will drop out of voluntary social health insurance? Evidence from the New Cooperative Medical Scheme in China**

Thank you for allowing me to review the resubmission. The resubmission indicates a manuscript that took into most of the suggestions.

I still have some reservations toward publication. I think the paper pays insufficient attention to the issues regarding determinants for dropping out of insurance. But these could be considered minor but important revision. I would like to see the paper again unless the editor is fine with the changes.

I believe the paper adds to how we should examine the determinants of outpatient visits and insurance enrollment (perhaps not insurance dropouts) although I have some questions about it which I elaborate on. I believe the results on dropout is less interesting in the authors treatment; Panda *et al.* (2016) had more interesting results.

Authors: In this paper, we focus first on the determinants of insurance dropouts, and second on the impact of drop-out on outpatient visits, not on the determinants of outpatient visits since the other determinants of outpatient visits except for dropout are regarded as covariates. We have now added to explanations on determinants of insurance dropouts in the discussion (see the 2^nd^ paragraph in Discussion).

I have two sets of comments to address:

(1) Dropping out: Usually, adverse selection has been associated with *perception* of illness rather than actual illness. Perception is affected by actual illnesses. It is strange to see that perception of health did not matter; it should be correlated with actual illness. It may be worth dropping the visits to doctors and chronic illness to see if perception matters. All three factors are correlated. I would suggest running all three together and each by itself. If I understand your results: I am more likely to drop out if I consider my health poorer than I consider my health excellent; but I am less likely to drop out if I visited health centers or have chronic illness. These results need to be examined, although the perceptions variables are insignificant the direction matters.

Authors: Thank you for the valuable suggestions. We have added suggested regressions by running all three factors together and each by itself. Results from running each by itself are consistent with the ones from running all three together. And yes, we agree with you that the direction of the perception variable matters though insignificant. We have revised accordingly in the result and discussion parts (see the 1^st^ paragraph in 4.1; Table 3; the 2^nd^ paragraph in Discussion).

It is also possible that poor health status is correlated with lower education and unemployment. Now, that I understand your work better, I find your result on drop-out needing much more explanation than what you note here. Retired people are more likely to have chronic illnesses; that they drop out seems odd. This goes against saying adverse selection is present. Your model is not sufficiently nuanced to explain some findings that may be contradictory. Vulnerable people simply may not be able to afford the insurance; I calculated the fee being about 10% of the Chinese poverty line. Examine if those economically vulnerable report poor or better health. Are the retired more likely to have visited a doctor? Examining how they are similar may help to disentangle some of your result. Adverse selection may not be the main factor driving dropouts

Authors: Yes, you are correct that poor health status may be correlated with lower education and unemployment. Therefore, we have controlled (included) variables indicating health status in the model when analyzing the impact of education/employment on drop-out. Results show that the retired people are more likely to drop out when they have the same number of chronic diseases with others (chronic diseases are controlled). Similarly, we have controlled health status in the model when examining the impact of economic status on drop-out, and controlled doctor visits when examining the impact of employment status on drop-out. And we agree with you that adverse selection may not be the main factor driving dropouts and we have added more explanations in the discussion part (see the 2^nd^ paragraph in Discussion).

(2) Visits to the Doctor: Thank you for clarifying the term DDD; I had not seen that used that way. You should be explicit that these are augmented DID models. Is Z having a chronic illness rather than not having one, as you do not have an estimation for b3 in case of chronic illness? I may be missing something here. The parallel trend model is helpful. I presume that is a negative binomial model. Those dropping out are less frequent user of health care, although insignificant.

Authors: We are sorry to confuse readers but as far as we know, augmented DID model is a complement to standard DID when the parallel trend assumption is violated (Li and Bell, 2017). Z denotes the number of chronic diseases, and we have the estimation for b_3_ in case of chronic illness. We do not show this result in the main text but in Appendix 5.

Yes, for the parallel trend model, it is negative binomial model. Please refer to the note in Appendix 2.

Things to mention in the interpretation:

(3) It is possible that the dropouts would have used health care less frequently prior to having insurance. That is, they are less users across time—a fixed effect. The test for parallel trend works against the first hypothesis of the paper.

Authors: Yes, the dropouts may use healthcare less frequently across time. And the first hypothesis of the paper when examining the determinants of drop-out is that people with less healthcare use in the previous years are more likely to drop out, which is not contradictory in our opinion. Please tell us if we misunderstand you at some point.

(4) I am skeptical of policy claims about health insurance effectiveness through measuring impact of dropouts. Dropouts are fundamentally different, perhaps completely unobserved, than those continuing to enrol. I think the result could be interesting if dropouts had significantly fewer visits prior to being insured at all. That could strengthen the hypothesis that adverse selection had an impact. If adverse selection is really the reason people drop out, then finding that they use less healthcare is to be expected.

Authors: This paper is trying to propose policy claims by measuring the impact of dropout on healthcare use, less about health insurance effectiveness. It is a very good suggestion to look at the doctor visits before being insured. But unfortunately, our sample only covers those who were enrolled in 2013, and we cannot get access to the data before being insured. We have edited the relevant part about adverse selection in the discussion (see the 2^nd^ paragraph in Discussion).

(5) Table 5 is interesting; that there is impact on secondary and tertiary care. But given your first set of results produce somewhat contradictory results, I am reluctant to say that lack of need for medical care is the fundamental reason people dropout.

Authors: Based on the first set of results, people who use less healthcare in the previous years are more likely to drop out. For people who use less healthcare, the reason for them to drop out is possibly lack of need for medical care. But for the other people, their reason may not be lack of need. As for the results shown in Table 5, it is not specifically for people who use less healthcare, but the average effect for the whole sample.

In short examine the first hypothesis in a more detailed way. And interpret the results of the second hypothesis cautiously toward policy conclusions regarding insurance in light of the fact that the first hypothesis does not produce clear results.

Authors: Thank you for the suggestions and we have revised accordingly.
