# Supplementary material for: Who will drop out of voluntary social health insurance? Evidence from the New Cooperative Medical Scheme in China
Source: Health Policy Plan. 2021 May 8;36(7):1013–22. doi: 10.1093/heapol/czab017 (PMC8530158; doi:10.1093/heapol/czab017)
Supplement: czab017_Supp [file czab017_supp.zip › Revised supplementary file.docx]

**Appendix 1.**

**The distribution of doctor visits for the drop-out group and the control group in 2013 and 2015**

**Figure 1. The density of doctor visits for the drop-out group and the control group in 2013 and 2015**

**Figure 2. The root of frequency of doctor visits for the drop-out group in 2013 and 2015**

**Figure 3. The density of doctor visits for the control group in 2013 and 2015**

**Appendix 2. Parallel trend test with data in 2011 and 2013**

|  | Doctor visits |
| --- | --- |
| Treatment * year | 0.908(0.118) |
| Treatment | 0.919(0.232) |
| Year, 1 (2011, ref) |  |
| Year, 2 (2013) | 1.258^***^(0.0582) |
| Equivalent income: quartile 1 (ref) |  |
| Equivalent income: quartile 2 | 1.102^*^(0.0633) |
| Equivalent income: quartile 3 | 1.122^*^(0.0710) |
| Equivalent income: quartile 4 | 1.120(0.0822) |
| Marital status: the married (ref) |  |
| Marital status: the single | 0.987(0.136) |
| No. of chronic diseases | 1.116^***^(0.0287) |
| Health status (ref: Excellent) |  |
| Very good | 1.463^*^(0.314) |
| Good | 2.175^***^(0.434) |
| Fair | 2.832^***^(0.569) |
| Poor | 3.235^***^(0.665) |
| Education attainment (ref: No education) |  |
| Elementary, middle school | 0.913(0.0921) |
| High school and above | 0.852(0.190) |
| Occupation (ref: Agricultural work) |  |
| Employed | 0.872(0.0822) |
| Self-employed | 1.087(0.113) |
| Retired/receded | 1.077(0.336) |
| Unemployed | 1.200^**^(0.0855) |
| Provincial GDP per capita | 0.902^**^(0.0379) |
| Constant | 0.168^***^(0.0448) |
| N | 19 780 |

Notes. Estimates stem from conditional fixed-effects negative binomial specifications. Coefficients represent incidence rate ratios. Standard errors are in parentheses. Significance levels: ***p<0.01; **p<0.05; *p<0.

**Appendix 3: Multivariate distance matching statistics**

|  | Matched | Total | Match rate |
| --- | --- | --- | --- |
| Treatment group | 730 | 751 | 97.2% |
| Control group | 9,695 | 9,881 | 98.1% |
| Combined | 10,425 | 10,632 | 98.1% |

**Appendix 4. Summary of standardized difference between the treatment and control group before and after matching**

|  | Raw |  |  | Matched |  |  |
| --- | --- | --- | --- | --- | --- | --- |
|  | Treatment group | Control group | SD | Treatment group | Control group | SD |
| Doctor visits | 0.417 | 0.516 | -0. 076 | 0.319 | 0.340 | 0.016 |
| Marital status: the married (ref) |  |  |  |  |  |  |
| Marital status: the single | 0.194 | 0.101 | 0.265 | 0.098 | 0.088 | 0.029 |
| Equivalent income | 10538.5 | 10621.7 | -0.004 | 8648.9 | 8948.4 | -0.013 |
| No. of chronic diseases | 1.220 | 1.280 | -0.047 | 1.091 | 1.200 | -0.081 |
| Self-perceived health status (ref: Excellent) |  |  |  |  |  |  |
| Very good | 0.097 | 0.110 | -0.040 | 0.093 | 0.099 | -0.017 |
| Good | 0.313 | 0.315 | -0.004 | 0.331 | 0.329 | 0.005 |
| Fair | 0.354 | 0.368 | -0.029 | 0.386 | 0.381 | 0.010 |
| Poor | 0.189 | 0.161 | 0.075 | 0.145 | 0.159 | -0.034 |
| Gender: male (ref) |  |  |  |  |  |  |
| Gender: female | 0.586 | 0.520 | 0.133 | 0.549 | 0.533 | 0.032 |
| Age | 61.5 | 59.0 | 0.264 | 59.7 | 59.2 | 0.052 |
| Provincial GDP per capita | 4.520 | 4.365 | 0.095 | 4.337 | 4.309 | 0.017 |
| Education attainment (ref: no education) |  |  |  |  |  |  |
| Elementary, middle school | 0.550 | 0.646 | -0.200 | 0.613 | 0.664 | -0.104 |
| High school and above | 0.037 | 0.068 | -0.138 | 0.061 | 0.040 | 0.097 |
| Occupation (ref: Agricultural Work) |  |  |  |  |  |  |
| Employed | 0.117 | 0.149 | -0.095 | 0.128 | 0.144 | -0.049 |
| Self-employed | 0.075 | 0.096 | -0.077 | 0.083 | 0.078 | 0.016 |
| Retired/receded | 0.020 | 0.015 | 0.041 | 0.012 | 0.007 | 0.037 |
| Unemployed | 0.298 | 0.199 | 0.231 | 0.195 | 0.201 | -0.013 |

Note. SD=Standardized difference. Results are from MDM kernel matching.

**Appendix 5.**

**Impacts of drop-out on outpatient care utilization at all health facilities**

|  | Incidence rate ratios of doctor visits | | |
| --- | --- | --- | --- |
|  | (1) | (2) | (3) |
| Treatment * year | 0.801^*^(0.108) | 1.192(0.272) | 0.376^***^(0.101) |
| Treatment | 0.748(0.201) | 0.410^**^(0.147) | 1.694(0.948) |
| Year, 1 (2013, ref) |  |  |  |
| Year, 2 (2015) | 1.201(0.158) | 1.496^*^(0.331) | 1.348(0.355) |
| Treatment * year * No. of chronic diseases |  | 0.735^***^(0.0801) |  |
| Treatment * No. of chronic diseases |  | 1.472^***^(0.204) |  |
| Year * No. of chronic diseases |  | 0.906(0.0939) |  |
| Treatment * year * 1.region |  |  | 2.805^**^(1.188) |
| Treatment * year * 2.region (ref) |  |  |  |
| Treatment * year * 3.region |  |  | 2.341^***^(0.754) |
| 1.region |  |  | 1.850(1.695) |
| 2.region (ref) |  |  |  |
| 3.region |  |  | 0.659(0.415) |
| Treatment * 1.region |  |  | 0.264(0.247) |
| Treatment * 2.region |  |  |  |
| Treatment * 3.region |  |  | 0.397(0.264) |
| Year * 1.region |  |  | 1.619(0.655) |
| Year * 2.region |  |  |  |
| Year * 3.region |  |  | 0.933(0.284) |
| Equivalent income: quartile 1 (ref) |  |  |  |
| Equivalent income: quartile 2 | 2.121^***^(0.155) | 2.223^***^(0.163) | 2.399^***^(0.179) |
| Equivalent income: quartile 3 | 1.398^***^(0.109) | 1.570^***^(0.127) | 1.499^***^(0.119) |
| Equivalent income: quartile 4 | 1.985^***^(0.168) | 2.409^***^(0.212) | 2.144^***^(0.186) |
| Marital status: the married (ref) |  |  |  |
| Marital status: the single | 1.080(0.205) | 1.225(0.239) | 1.024(0.208) |
| No. of chronic diseases | 1.291^***^(0.0470) | 1.143(0.151) | 1.322^***^(0.0499) |
| Health status (ref: Excellent) |  |  |  |
| Very good | 0.113^***^(0.0265) | 0.134^***^(0.0325) | 0.123^***^(0.0296) |
| Good | 0.334^***^(0.0664) | 0.460^***^(0.0948) | 0.361^***^(0.0731) |
| Fair | 0.249^***^(0.0509) | 0.301^***^(0.0637) | 0.288^***^(0.0602) |
| Poor | 0.386^***^(0.0799) | 0.463^***^(0.0990) | 0.415^***^(0.0875) |
| Education attainment (ref: No education) |  |  |  |
| Elementary, middle school | 0.976(0.115) | 0.999(0.120) | 1.222(0.149) |
| High school and above | 1.652^***^(0.303) | 1.615^**^(0.306) | 1.692^***^(0.341) |
| Occupation (ref: Agricultural work) |  |  |  |
| Employed | 0.550^***^(0.0652) | 0.508^***^(0.0615) | 0.551^***^(0.0670) |
| Self-employed | 0.488^***^(0.0499) | 0.493^***^(0.0515) | 0.476^***^(0.0496) |
| Retired/receded | 0.134(0.176) | 0.148(0.194) | 0.156(0.209) |
| Unemployed | 0.484^***^(0.0405) | 0.467^***^(0.0394) | 0.483^***^(0.0417) |
| Provincial GDP per capita | 0.872^***^(0.0423) | 0.853^***^(0.0414) | 0.653^***^(0.0855) |
| Constant | 1.487(0.572) | 1.484(0.659) | 4.088^*^(3.327) |
| n | 22 982 | 22 982 | 22 982 |

Notes. Estimates stem from conditional fixed-effects negative binomial specifications. Coefficients represent incidence rate ratios. Standard errors are in parentheses. Significance levels: ***p<0.01; **p<0.05; *p<0.1.

**Appendix 6.**

**Impacts of drop-out on outpatient care utilization at different levels of health facilities**

|  | Incidence rate ratios of doctor visits | |
| --- | --- | --- |
|  | Primary care clinics | Secondary and tertiary hospitals |
|  | (1) | (2) |
| Treatment * year | 1.065(0.189) | 0.617^**^(0.146) |
| Treatment | 0.496^**^(0.171) | 1.595(0.909) |
| Year, 1 (2013, ref) |  |  |
| Year, 2 (2015) | 1.096(0.190) | 1.299(0.297) |
| Equivalent income: quartile 1 (ref) |  |  |
| Equivalent income: quartile 2 | 1.833^***^(0.178) | 2.441^***^(0.314) |
| Equivalent income: quartile 3 | 1.351^***^(0.142) | 1.688^***^(0.220) |
| Equivalent income: quartile 4 | 1.682^***^(0.190) | 1.486^***^(0.219) |
| Marital status: the married (ref) |  |  |
| Marital status: the single | 1.545(0.410) | 0.661(0.238) |
| No. of chronic diseases | 1.216^***^(0.0565) | 1.429^***^(0.111) |
| Health status (ref: Excellent) |  |  |
| Very good | 0.108^***^(0.0447) | 0.230^***^(0.0774) |
| Good | 0.464^**^(0.171) | 0.361^***^(0.108) |
| Fair | 0.301^***^(0.111) | 0.273^***^(0.0856) |
| Poor | 0.302^***^(0.113) | 0.490^**^(0.153) |
| Education attainment (ref: No education) |  |  |
| Elementary, middle school | 0.959(0.149) | 0.814(0.197) |
| High school and above | 1.896^*^(0.640) | 1.377(0.416) |
| Occupation (ref: Agricultural work) |  |  |
| Employed | 0.749^*^(0.121) | 0.485^***^(0.0985) |
| Self-employed | 0.231^***^(0.0465) | 0.697^**^(0.106) |
| Retired/receded | 0.255(0.744) | 0.0793(0.138) |
| Unemployed | 0.371^***^(0.0451) | 0.710^**^(0.0989) |
| Provincial GDP per capita | 0.874^*^(0.0707) | 0.932(0.0731) |
| Constant | 0.930 (0.555) | 0.320(0.226) |
| n | 22 982 | 22 982 |

Notes. Estimates stem from conditional fixed-effects negative binomial specifications. Coefficients represent incidence rate ratios. Standard errors are in parentheses. Significance levels: ***p<0.01; **p<0.05; *p<0.

**Appendix 7. Robustness checks with equivalent expenditure**

|  | Odds ratios of drop-out | Impacts at all health facilities |
| --- | --- | --- |
|  | (1) | (2) |
| Treatment * year |  | 0.790^*^(0.108) |
| Treatment |  | 0.942(0.254) |
| Year, 1 (2013, ref) |  |  |
| Year, 2 (2015) |  | 1.232(0.164) |
| Doctor visits in 2013 | 0.935^**^(0.0301) |  |
| Equivalent expenditure: quartile 1 (ref) |  |  |
| Equivalent expenditure: quartile 2 | 0.812^*^(0.0906) | 2.829^***^(0.230) |
| Equivalent expenditure: quartile 3 | 0.699^***^(0.0803) | 2.485^***^(0.217) |
| Equivalent expenditure: quartile 4 | 0.830(0.108) | 2.915^***^(0.280) |
| No. of chronic diseases | 0.882^***^(0.0314) | 1.242^***^(0.0452) |
| Health status (ref: Excellent) |  |  |
| Very good | 1.219(0.291) | 0.105^***^(0.0242) |
| Good | 1.080(0.218) | 0.351^***^(0.0704) |
| Fair | 1.284(0.265) | 0.285^***^(0.0587) |
| Poor | 1.381(0.309) | 0.425^***^(0.0893) |
| Education attainment (ref: No education) |  |  |
| Elementary, middle school | 0.732^***^(0.0699) | 0.966(0.114) |
| High school and above | 0.620^***^(0.0918) | 1.919^***^(0.336) |
| Occupation (ref: Agricultural work) |  |  |
| Employed | 0.709^**^(0.108) | 0.572^***^(0.0672) |
| Self-employed | 1.287^*^(0.181) | 0.513^***^(0.0508) |
| Retired/receded | 1.890^**^(0.506) | 1.529(0.881) |
| Unemployed | 1.325^***^(0.129) | 0.605^***^(0.0500) |
| Marital status: the married (ref) |  |  |
| Marital status: the single | 1.597^***^(0.187) | 0.972(0.197) |
| Age | 1.013^**^(0.00632) |  |
| Gender: male (ref) |  |  |
| Gender: female | 1.047(0.0835) |  |
| Region: 1 | 2.054^***^(0.359) |  |
| Region: 2 (ref) |  |  |
| Region: 3 | 1.510^***^(0.210) |  |
| Provincial GDP per capita |  | 0.839^***^(0.0396) |
| Constant | 0.0301^***^(0.0132) | 1.046(0.402) |
| n | 11 491 | 22 982 |

Notes. Estimates stem from logistic specifications and conditional fixed-effects negative binomial specifications. Standard errors in column 1 are clustered on villages (listed in parentheses). Coefficients in column 2 represent incidence rate ratios. Significance levels: ***p<0.01; **p<0.05; *p<0.1.

**Appendix 8. Odds ratios of non-enrollment in the NCMS between 2013 and 2015**

|  | Odds ratios/standard errors |
| --- | --- |
| Doctor visits | 0.998(0.0413) |
| Equivalent income (ref: Quartile 1) |  |
| Quartile 2 | 0.823(0.120) |
| Quartile 3 | 0.973(0.152) |
| Quartile 4 | 1.010(0.167) |
| No. of chronic diseases | 0.862^*^(0.0741) |
| Self-perceived health status (ref: Excellent) |  |
| Very good | 1.344(0.399) |
| Good | 1.033(0.306) |
| Fair | 1.144(0.342) |
| Poor | 1.142(0.377) |
| Education attainment (ref: No education) |  |
| Elementary, middle school | 1.344(0.583) |
| High school and above | 1.786(0.951) |
| Occupation (ref: Agricultural work) |  |
| Employed | 0.764(0.169) |
| Self-employed | 1.396(0.312) |
| Retired/receded | 0.453(0.252) |
| Unemployed | 1.384^*^(0.237) |
| Marital status (ref: The married) |  |
| The single | 0.762(0.290) |
| Provincial GDP per capita | 4.323^***^(0.577) |
| n | 23 948 |

Notes. Estimates are derived from conditional fixed-effects logistic regression models. Standard errors are listed in parentheses. Significance levels: ***p<0.01; **p<0.05; *p<0.1.

**Appendix 9. Odds ratios of dropping out from the NCMS in 2015-clustered on household**

|  | Odds ratios of drop-out/standard errors |
| --- | --- |
| Doctor visits in 2013 | 0.933^**^(0.0286) |
| Equivalent income (ref: Quartile 1) |  |
| Quartile 2 | 0.780^**^(0.0911) |
| Quartile 3 | 0.810^*^(0.0972) |
| Quartile 4 | 0.931(0.114) |
| No. of chronic diseases | 0.883^***^(0.0285) |
| Self-perceived health status (ref: Excellent) |  |
| Very good | 1.215(0.264) |
| Good | 1.099(0.211) |
| Fair | 1.312(0.251) |
| Poor | 1.426^*^(0.294) |
| Education attainment (ref: No education) |  |
| Elementary, middle school | 0.732^***^(0.0681) |
| High school and above | 0.609^***^(0.0879) |
| Occupation (ref: Agricultural work) |  |
| Employed | 0.707^**^(0.100) |
| Self-employed | 1.251(0.182) |
| Retired/receded | 1.829^**^(0.508) |
| Unemployed | 1.281^**^(0.126) |
| Marital status (ref: The married) |  |
| The single | 1.645^***^(0.179) |
| Age | 1.016^***^(0.00549) |
| Gender (ref: The male) |  |
| The female | 1.054(0.0859) |
| Region (ref: The middle provinces) |  |
| The richest provinces | 1.972^***^(0.241) |
| The poorest provinces | 1.506^***^(0.165) |
| Constant | 0.0233^***^(0.00958) |
| n | 11 491 |

Notes. Estimates are derived from logistic regression models. Standard errors are clustered on household (listed in parentheses). Significance levels: ***p<0.01; **p<0.05; *p<0.1.
