# Supplementary material for: Who will drop out of voluntary social health insurance? Evidence from the New Cooperative Medical Scheme in China
Source: Health Policy Plan. 2021 May 8;36(7):1013–22. doi: 10.1093/heapol/czab017 (PMC8530158; doi:10.1093/heapol/czab017)
Supplement: czab017_Supp [file czab017_supp.zip › Tables-revised 190121 (highlight).docx]

**Table 1. The number of individuals with each insurance type across two waves for 15 669 individuals enrolled in both CHARLS 2013 and 2015**

| 2015  2013 | UEBMI | URBMI | NCMS | URRMI | GMI | None | Total (%) |
| --- | --- | --- | --- | --- | --- | --- | --- |
| UEBMI | 1181 | 89 | 43 | 9 | 208 | 137 | 1667  (10.6) |
| URBMI | 105 | 419 | 87 | 29 | 15 | 74 | 729  (4.7) |
| **NCMS** | **82** | **107** | **10 718** | **267** | **52** | **879** | **12 105**  **(77.3)** |
| URRMI | 9 | 45 | 214 | 22 | 2 | 24 | 316  (2.0) |
| GMI | 111 | 8 | 11 | 2 | 110 | 17 | 259  (1.7) |
| None | 28 | 46 | 283 | 10 | 15 | 211 | 593  (3.8) |
| Total (%) | 1516  (9.7) | 714  (4.6) | 11 356  (72.5) | 339  (2.2) | 402  (2.6) | 1342  (8.6) | 15 669 |

**Table2. Descriptive statistics of 22 982 observations in 2013 and 2015**

|  | 2013, Mean (SD) | | 2015, Mean (SD) | |
| --- | --- | --- | --- | --- |
|  | Drop-out group | Control group | Drop-out group | Control group |
| Doctor visits |  |  |  |  |
| All health facilities | 0.42 (1.07), | 0.52 (1.54) | 0.38 (1.30) | 0.47 (1.52) |
| Secondary & tertiary hospitals | 0.14 (0.61) | 0.13 (0.67) | 0.14 (0.78) | 0.13 (0.67) |
| Primary care clinics | 0.24 (0.82) | 0.34 (1.32) | 0.22 (0.96) | 0.31 (1.28) |
| Equivalent income, RMB |  |  |  |  |
| Quartile (25%) | 587.9 | 750.6 | 0 | 115.5 |
| Quartile (50%) | 2840.6 | 4780 | 1000 | 1500 |
| Quartile (75%) | 12 990.4 | 14 433.8 | 9263.1 | 11 547.0 |
| Marital status, % |  |  |  |  |
| The married | 77.2 | 88.4 | 75.4 | 86.6 |
| The single | 22.8 | 11.6 | 24.6 | 13.4 |
| No. of chronic diseases | 1.22 (1.29) | 1.28 (1.33) | 1.27 (1.31) | 1.41 (1.41) |
| Self-perceived health status, % |  |  |  |  |
| Excellent | 4.45 | 4.69 | 5.04 | 5.96 |
| Very good | 9.26 | 10.9 | 11.1 | 9.97 |
| Good | 31.9 | 31.1 | 26.7 | 30.3 |
| Fair | 34.9 | 36.8 | 37.2 | 36.2 |
| Poor | 19.5 | 16.6 | 20.0 | 17.6 |
| Age | 62.2 (10.6) | 59.5 (9.39) | 64.2 (10.7) | 61.4 (9.39) |
| Gender, % |  |  |  |  |
| Male | 40.6 | 46.9 | 40.6 | 46.9 |
| Female | 59.4 | 53.1 | 59.4 | 53.1 |
| Education attainment, % |  |  |  |  |
| No education | 43.6 | 29.9 | 43.2 | 30.0 |
| Elementary, middle school | 52.5 | 63.4 | 46.7 | 54.6 |
| High school and above | 3.86 | 6.67 | 10.2 | 15.4 |
| Occupation |  |  |  |  |
| Agricultural work | 48.4 | 53.6 | 44.8 | 47.7 |
| Employed | 11.5 | 15.0 | 9.41 | 17.2 |
| Self-employed | 7.45 | 9.39 | 8.52 | 8.38 |
| Retired/receded | 2.02 | 1.45 | 2.04 | 1.34 |
| Unemployed | 30.7 | 20.6 | 35.2 | 25.3 |
| Prop. of drop-out, % | 7.44 | | | |
| n | n=855 | n=10 636 | n=855 | n=10 636 |

Notes. SD=Standard deviation (in parentheses).

**Table 3. Odds ratios of dropping out from the NCMS in 2015**

|  | Odds ratios of drop-out/standard errors | | | |
| --- | --- | --- | --- | --- |
|  | (1) | (2) | (3) | (4) |
| No. of doctor visits in 2013 | 0.933**(0.0300) | 0.923**(0.0308) |  |  |
| Equivalent income (ref: Quartile 1) |  |  |  |  |
| Quartile 2 | 0.780^**^(0.0936) | 0.798^*^(0.0965) | 0.766^**^(0.0928) | 0.814^*^(0.0967) |
| Quartile 3 | 0.810^*^(0.103) | 0.825(0.105) | 0.803^*^(0.101) | 0.847(0.107) |
| Quartile 4 | 0.931(0.117) | 0.950(0.119) | 0.909(0.114) | 0.943(0.118) |
| No. of chronic diseases | 0.883^***^(0.0314) |  |  | 0.889^***^(0.0313) |
| Self-perceived health status (ref: Excellent) |  |  |  |  |
| Very good | 1.215(0.288) |  | 1.175(0.259) |  |
| Good | 1.099(0.222) |  | 0.979(0.189) |  |
| Fair | 1.312(0.271) |  | 1.118(0.224) |  |
| Poor | 1.426(0.319) |  | 1.137(0.244) |  |
| Education attainment (ref: No education) |  |  |  |  |
| Elementary, middle school | 0.732^***^(0.0703) | 0.722^***^(0.0689) | 0.727^***^(0.0682) | 0.721^***^(0.0688) |
| High school and above | 0.609^***^(0.0922) | 0.593^***^(0.0887) | 0.600^***^(0.0906) | 0.579^***^(0.0875) |
| Occupation (ref: Agricultural work) |  |  |  |  |
| Employed | 0.707^**^(0.111) | 0.682^**^(0.106) | 0.710^**^(0.111) | 0.661^***^(0.102) |
| Self-employed | 1.251(0.180) | 1.211(0.172) | 1.217(0.173) | 1.183(0.167) |
| Retired/receded | 1.829^**^(0.496) | 1.765^**^(0.477) | 1.783^**^(0.483) | 1.747^**^(0.472) |
| Unemployed | 1.281^**^(0.126) | 1.269^**^(0.121) | 1.210^*^(0.118) | 1.296^***^(0.124) |
| Marital status (ref: The married) |  |  |  |  |
| The single | 1.645^***^(0.195) | 1.662^***^(0.196) | 1.679^***^(0.193) | 1.692^***^(0.198) |
| Age | 1.016^**^(0.00643) | 1.015^**^(0.00606) | 1.014^**^(0.00633) | 1.015^**^(0.00608) |
| Gender (ref: The male) |  |  |  |  |
| The female | 1.054(0.0849) | 1.011(0.0786) | 1.025(0.0803) | 1.018(0.0791) |
| Region (ref: The middle provinces) |  |  |  |  |
| The richest provinces | 1.972^***^(0.339) | 1.998^***^(0.333) | 2.020^***^(0.341) | 1.973^***^(0.330) |
| The poorest provinces | 1.506^***^(0.210) | 1.493^***^(0.205) | 1.504^***^(0.205) | 1.467^***^(0.198) |
| Constant | 0.0233^***^(0.0104) | 0.0267^***^(0.0112) | 0.0261^***^(0.0116) | 0.0303^***^(0.0126) |
| n | 11 491 | 11 491 | 11 491 | 11 491 |

Notes. Estimates are derived from logistic regression models. Standard errors are clustered on villages (listed in parentheses). Significance levels: ***p<0.01; **p<0.05; *p<0.1.

**Table 4. Impacts of drop-out on outpatient care utilization at all health facilities**

|  | Incidence rate ratios of doctor visits | | |
| --- | --- | --- | --- |
|  | (1) | (2) | (3) |
| Treatment * year | 0.801^*^(0.108) | 1.192(0.272) | 0.376^***^(0.101) |
| Treatment | 0.748(0.201) | 0.410^**^(0.147) | 1.694(0.948) |
| Year, 1 (2013, ref) |  |  |  |
| Year, 2 (2015) | 1.201(0.158) | 1.496^*^(0.331) | 1.348(0.355) |
| Treatment * year * No. of chronic diseases |  | 0.735^***^(0.0801) |  |
| Treatment * No. of chronic diseases |  | 1.472^***^(0.204) |  |
| Year * No. of chronic diseases |  | 0.906(0.0939) |  |
| Treatment * year * 1.region |  |  | 2.805^**^(1.188) |
| Treatment * year * 2.region (ref) |  |  |  |
| Treatment * year * 3.region |  |  | 2.341^***^(0.754) |
| Treatment * 1.region |  |  | 0.264(0.247) |
| Treatment * 2.region (ref) |  |  |  |
| Treatment * 3.region |  |  | 0.397(0.264) |
| Year * 1.region |  |  | 1.619(0.655) |
| Year * 2.region (ref) |  |  |  |
| Year * 3.region |  |  | 0.933(0.284) |
| Covariates | Yes | Yes | Yes |
| n | 22 982 | 22 982 | 22 982 |

Notes. Estimates stem from conditional fixed-effects negative binomial specifications. Coefficients represent incidence rate ratios. Standard errors are in parentheses. Significance levels: ***p<0.01; **p<0.05; *p<0.1.

**Table 5. Impacts of drop-out on outpatient care utilization at different health facilities**

|  | | Incidence rate ratios of doctor visits | |
| --- | --- | --- | --- |
|  | Primary care clinics | | Secondary and tertiary hospitals |
|  | (1) | | (2) |
| Treatment * year | 1.065(0.189) | | 0.617^**^(0.146) |
| Treatment | 0.496^**^(0.171) | | 1.595(0.909) |
| Year, 1 (2013, ref) |  | |  |
| Year, 2 (2015) | 1.096(0.190) | | 1.299(0.297) |
| Covariates | Yes | | Yes |
| n | 22 982 | | 22 982 |

Notes. Estimates stem from conditional fixed-effects negative binomial specifications. Coefficients represent incidence rate ratios. Standard errors are in parentheses. Significance levels: ***p<0.01; **p<0.05; *p<0.1.
